# 33 Targeting Vulnerability in the Homeless–A National Analysis of Burn Injuries Presenting to the Emergency Department.

**DOI:** 10.1093/jbcr/irad045.007

**Published:** 2023-05-15

**Authors:** Jennifer Shah, Farrah Liu, Priscila Cevallos, Uchechukwu Amakiri, Thomas Johnstone, Clifford Sheckter, Rahim Nazerali

**Affiliations:** Stanford University, New York, New York; Stanford University, Stanford, California; Stanford University, Stanford, California; Icahn School of Medicine at Mount Sinai, New York, New York; Stanford University, Stanford, California; Stanford University, Palo Alto, California; Stanford University, Stanford, California; Stanford University, New York, New York; Stanford University, Stanford, California; Stanford University, Stanford, California; Icahn School of Medicine at Mount Sinai, New York, New York; Stanford University, Stanford, California; Stanford University, Palo Alto, California; Stanford University, Stanford, California; Stanford University, New York, New York; Stanford University, Stanford, California; Stanford University, Stanford, California; Icahn School of Medicine at Mount Sinai, New York, New York; Stanford University, Stanford, California; Stanford University, Palo Alto, California; Stanford University, Stanford, California; Stanford University, New York, New York; Stanford University, Stanford, California; Stanford University, Stanford, California; Icahn School of Medicine at Mount Sinai, New York, New York; Stanford University, Stanford, California; Stanford University, Palo Alto, California; Stanford University, Stanford, California; Stanford University, New York, New York; Stanford University, Stanford, California; Stanford University, Stanford, California; Icahn School of Medicine at Mount Sinai, New York, New York; Stanford University, Stanford, California; Stanford University, Palo Alto, California; Stanford University, Stanford, California; Stanford University, New York, New York; Stanford University, Stanford, California; Stanford University, Stanford, California; Icahn School of Medicine at Mount Sinai, New York, New York; Stanford University, Stanford, California; Stanford University, Palo Alto, California; Stanford University, Stanford, California; Stanford University, New York, New York; Stanford University, Stanford, California; Stanford University, Stanford, California; Icahn School of Medicine at Mount Sinai, New York, New York; Stanford University, Stanford, California; Stanford University, Palo Alto, California; Stanford University, Stanford, California

## Abstract

**Introduction:**

Burn management is complex and outcomes depend on injury severity, timing of presentation, and specialized multidisciplinary care. Burn injuries among the homeless are an increasing problem in the United States (US) as record numbers of people are unsheltered and using unsafe heating practices. This study aims to characterize mechanism, demographic, and social factors related to burn injuries in homeless persons.

**Methods:**

Burn encounters were extracted from the 2019 Nationwide Emergency Department Sample (NEDS) database. Two cohorts were created comparing homeless and non-homeless encounters. Burn characteristics (size, depth, location and mechanism), comorbidities, demographics, and ED treatments were compared with univariate testing. Multivariable regression evaluated the likelihood of admission. Discharge weights were used to yield national estimates.

**Results:**

Of 316,334 ED visits meeting criteria for burn injuries in 2019, 1,919 (0.6%) had a diagnosis code for homelessness. Homelessness burn encounters were significantly older (mean age 32.4 vs. 44.8), were more often male (71% vs. 52%), were more often White (59% vs. 54%) or Black (24% vs. 21%), were more often presenting to EDs in the Western region of the US (43% vs. 19%), and more often had Medicaid as primary payer (51% vs 33%). Burns in homeless encounters more commonly resulted from flame injury and more commonly involved the lower extremity (p< 0.001). Among homeless encounters, burn injuries were more often due to self-harm (12% vs. 2%) or assault (5% vs. 1%)(p< 0.001). In addition, homeless encounters had significantly greater mental illness related to substance abuse (69% vs 16%, p< 0.001) and had greater comorbidity burden (38% vs. 6% had Elixhauser index scores of 3 or higher; p< 0.001). Homeless encounters experienced higher rates of third degree burns and concomitant injuries (p≤0.003). 49% vs 7% of homeless and non-homeless encounters, respectively, required admission, and homelessness was associated with higher odds of admission (adjusted OR 4.58; 95% CI 3.066–6.828; p< 0.001).

**Conclusions:**

Burn injuries in the homeless affect a vulnerable population who is older, has more comorbidities, and has deeper burns. Mental illness is over 4 times more likely in this population who is also at increased risk of getting burned through assault.

**Applicability of Research to Practice:**

Burn injuries presenting to the ED should be screened for homelessness given the associated risk factors. Legal, medical, and mental health resources can then be appropriately targeted.

